# RETRACTION: Effects of Osteocalcin on Synthesis of Testosterone and INSL3 during Adult Leydig Cell Differentiation

**DOI:** 10.1155/ije/9796454

**Published:** 2026-04-28

**Authors:** International Journal of Endocrinology

RETRACTION: G. Coskun, L. Sencar, A. Tuli, D. Saker, M. M. Alparslan, and S. Polat, “Effects of Osteocalcin on Synthesis of Testosterone and INSL3 during Adult Leydig Cell Differentiation,” *International Journal of Endocrinology*, no. 2019 (2019), https://doi.org/10.1155/2019/1041760.

The above article, published online on 28 August 2019 in Wiley Online Library (https://wileyonlinelibrary.com), has been retracted following the agreement of the journal’s Chief Editor, Zhongjian Xie; and John Wiley & Sons Ltd.

The retraction has been agreed following an investigation of the concerns raised by a third party on PubPeer [1], which identified multiple instances of inappropriately overlapping figures. Our investigation concluded that there are concerns about the reliability of this article as follows:

- Figure 3h: The testicular tissue shown in the figure appears similar to Figure 2a of [2], where it is described as part of a different experiment.
- Figure 4c: The Leydig cells appear identical to the cells shown in Figure 5c, which are attributed to a different treatment group.
- Figure 4h: The images of the Leydig cells share overlap with Figure 5h, however are attributed to different experimental groups.
- Figure 4j: The images of the Leydig cells have overlapping features with the cells presented in Figure 4k, despite representing tissues from different animals and experimental conditions.
- Figure 4l: Concerns have been raised regarding the similarity of the depicted cells to Figure 4d of a subsequent paper by the listed authors [3].
- Figure 9a: The electron microscopy image contains multiple unusually overlapping features, raising concerns about its authenticity.

As a result of the investigation, the results of the article are considered unreliable and it is therefore retracted.

The authors disagree with this retraction.
